# Silencing of Rieske Iron-Sulfur Protein Impacts Upon the Development and Reproduction of *Spodoptera exigua* by Regulating ATP Synthesis

**DOI:** 10.3389/fphys.2018.00575

**Published:** 2018-05-24

**Authors:** Song Shi, Hongliang Zuo, Lu Gao, Xin Yi, Guohua Zhong

**Affiliations:** ^1^Key Laboratory of Crop Integrated Pest Management in South China, Ministry of Agriculture, South China Agricultural University, Guangzhou, China; ^2^Key Laboratory of Natural Pesticide and Chemical Biology, Ministry of Education, South China Agricultural University, Guangzhou, China

**Keywords:** rieske iron-sulfur protein, ATP synthesis, *Spodoptera exigua*, developmental inhibition, RNAi

## Abstract

Rieske iron-sulfur protein (RISP) is a key protein subunit of mitochondrial complex III which plays an important role in the respiratory electron transport chain. The complete cDNA of RISP was cloned from *Spodoptera exigua* by real time quantitative PCR and rapid-amplification of cDNA ends (RACE) technology and named as *SeRISP* (GenBank Accession Number: JN992290). Multiple alignments and the creation of a phylogenetic tree revealed that RISPs are highly conserved among different insects, and the highly conserved region of RISPs is mainly located at the C-terminal which serves as the functional domain. Expression pattern analysis demonstrated that *SeRISP* is expressed in all developmental stages of *S. exigua*; the expression levels increased during larval growth, remained stable during development from fourth instar to pupa and reached a peak in the adult. In addition, *SeRISP* was significantly suppressed at both the mRNA and protein levels by feeding the instar stage with dsRNA; levels of suppression increased with increasing dsRNA concentration and continuous treatment time. The silencing of SeRISP in larvae led to the significant inhibition of ATP synthesis and larval growth, which could result in energy reserve deficiency in pupae and the suppression of fecundity and hatchability in adults. Our findings confirmed that it is possible to silence target genes in *S. exigua* by simple dsRNA feeding, and provided evidence of the essential role of RISP in the process of ATP synthesis, growth and reproduction.

## Introduction

Mitochondria are vital for an extensive number of cellular processes, yet hundreds of proteins involved in this process lack precise identification and robust functional annotation (Floyd Brendan et al., 2016; Li et al., 2017). As the principal organelle responsible for metabolism, mitochondria participate in a wide range of functions related to cellular metabolism (Floyd Brendan et al., 2016). Protein complex III, consisting of cytochrome c1, two forms of cytochrome b (b562 and b566) and a rieske iron-sulfur protein (RISP) containing a high potential rieske [2Fe-2S] cluster, is known to play an important role in the function of ATP synthase (Moghaddam et al., 2017). It does this, by generating an electrochemical potential on both sides of the mitochondrial inner membrane for ATP synthesis (Heinrich et al., 2017). In insect, ATP synthase is a large multi-protein complex; defects in this complex could lead to deficiencies in ATP production and overall mitochondrial dysfunction, and eventually lead to neuromuscular disorders (Sawyer et al., 2017). Consequently, the α-subunit of ATP synthase could be considered as a potential target for insect pest control (Hu and Xia, 2016).

In *Caenorhabdities elegans*, the mutation of the highly conserved rieske iron-sulfur subunit of complex III in the respiratory chain results in pleiotropic phenotypes, including delayed development and increased lifespan (Jafari et al., 2015). In the field of entomological physiology, the structural and functional characteristics of [2Fe-2S] have been widely investigated in insects because of its critical role in the respiratory chain. The structure of the [2Fe-2S] cluster in *Spodoptera littoralis* was first revealed by electron paramagnetic resonance (EPR), which showed distinct signals compared to those associated with mitochondrial NADH dehydrogenase and succinate dehydrogenase; this difference was attributed to the presence of ferredoxin (Ali et al., 2017; Emam et al., 2017). And those results provide important information for further research on respiratory chain in insects (Shergill et al., 1995). The silencing of RISP in cell lines of *Trypanosoma brucei* by dsRNA transfection led to the significant suppression of respiration and metabolism, thus providing evidence for the essential role of RISP in the life cycle of *T. brucei* (Smíd et al., 2006). Similarly, in *Plutella xylostella* larva, there was a close association between RISP levels and ATP content (Gong et al., 2011). As a crucial electron mediator in the respiratory chain, the suppression of RISP can inhibit the respiratory chain, reduce the synthesis of ATP and subsequently inhibit the development of insects.

RISP has not only been identified in model insects in which the entire genome has been sequenced, such as *Bombyx mori, Tribolium castaneum, Drosophila melanogaster, Aedes aegypti*, and *Anopheles gambiae* (Holt et al., 2002; Hoskins et al., 2007; Nene et al., 2007; Kim et al., 2009), but has also been identified in some non-model insects, such as *P. xylostella, Graphocephala atropunctata*, and *Culex quinquefasciatus* (Gong et al., 2011; Li and Xia, 2012). *Spodoptera exigua* is a prevalent agricultural pest in China which causes enormous economic loss from agriculture. Unfortunately, this pest has also developed resistance to many broad-spectrum insecticides, because of the long-term and unreasonable utilization of chemical pesticides (Jia et al., 2009; Lai and Su, 2011). Under these circumstances, the development and application of a sustainable, efficient and safe method to control this pest is becoming increasingly important. In the present study, we characterize the gene/protein structure and expression pattern of RISP in *S. exigua* (referred to hereafter as SeRISP). In addition, we described the effects of silencing SeRISP by feeding *S. exigua* larvae with dsRNA during post-embryonic development. Our findings provide a foundation for a better understanding of RISP function in the process of ATP synthesis, larval growth and adult reproduction.

## Materials and methods

### Insects

A laboratory strain of *S. exigua* was reared in the Key Laboratory of Crop Integrated Pest Management in South China, Ministry of Agriculture, Guangzhou, China. Insects were routinely reared at 26 ± 1°C and 70 ± 10% relative humidity under a 16:8 h (light:dark) photoperiod.

### RNA extraction and reverse transcription

Total RNA was extracted from all stages of *S. exigua* using a Total RNA kit II (Omega Bio-tek, Norcross, GA) according to the manufacturer's instructions. cDNA was synthesized from total RNA with an Oligo d(T)_18_ primer. Reverse transcriptase reactions contained 1 μg of RNA samples, 1 μL of 50 μM Oligo d(T)_18_ primer, 1 μL of 10 mM of each dNTP (TaKaRa, Dalian, China), 1 μL of 200 U·μL^−1^ PrimerScript Reverse Transcriptase (TaKaRa, Dalian, China) and 0.5 μL of 40 U·μL^−1^ RNase Inhibitor (TaKaRa, Dalian, China). 10 μL reactions, consisting of total RNA, Oligo d(T)_18_ primer and ddH_2_O were first incubated for 5 min at 65°C, followed by 2 min in an ice-bath. The remaining reagents were then added following centrifugation. The final 25 μL reactions were incubated in a MyCycler Thermal Cycler (Bio-Rad, Hercules, CA), for 60 min at 42°C, 15 min at 70°C and then held at 4°C.

### Cloning and sequencing of seRISP

Degenerate primers, SeRISPDF and SeRISPDR (Table 1), were designed based on the homologous regions in the amino acid sequence of RISP from *B. mori* (GenBank Accession Number: NM 001113267.1) and *S. litura* (GenBank Accession Number: HQ599193.1) (Chen et al., 2011) and used to clone a partial sequence of the RISP gene from *S. exigua* (*SeRISP*) cDNA. Touch-down polymerase chain reaction (PCR) was used to clone the partial sequence of *SeRISP*, and was performed in a 50 μL reaction volume containing 1 μL of cDNA, 1 μL of 10 μM of each primer, 5 μL of 10 × PCR buffer, 4 μL of 2.5 mM of each dNTP (TaKaRa, Dalian, China) and 0.5 of μL 5 U·μL^−1^ *Ex* Taq DNA polymerase (TaKaRa, Dalian, China). The PCR cycle was 94°C for 3 min, followed by 10 cycles of 94°C 30 s, an annealing temperature decrease of 55–50°C for 30 s (including a 0.5°C reduction in temperature for each cycle), 72°C for 1 min; then 25 cycles of 94°C for 30 s, 50°C for 30 s, 72°C for 1 min, and a final polymerization step at 72°C for 10 min. Target fragments were purified from 2.0% agarose gel, ligated into the T-vector (TaKaRa, Dalian, China) and then sequenced.

**Table 1:** Primers used in the experiment.

**Function**	**Name**	**Sequence (5^′^-3^′^)**
Degenerate primers	SeRISPDF	GCTGGGCATTTGGCYCCTTA
	SeRISPDR	GGAKCCRTGGCAGGGCAGTA
3′ RACE	SeRISP3EF1	TGGTAAGCCACTGTTCATCCG
	SeRISP3EF2	TAAGTGGCTGGTAGTTATTGGTG
	3′ RACE outer primer	TACCGTCGTTCCACTAGTGATTT
	3′ RACE inner primer	CGCGGATCCTCCACTAGTGATTTCACTATAGG
5′ RACE	SeRISP5ER1	AGCCAAAGCCAAGACATCAGC
	SeRISP5ER2	GCTCCAGCAATGAGATAGGTGA
	SeRISP5ER3	TCGGTTCCTGGGTCTCCTTGC
	AUAP primer	GGCCACGCGTCGACTAGTAC
	Abridged anchor primer	GGCCACGCGTCGACTAGTACGGGIGGGIIGGGIIG
qRT-PCR	SeRISPRTF	TGCTGATGTCTTGGCTTTGG
	SeRISPRTR	CTGCTCGGTTGAAATCTCGTT
	SeActRTF	CGTCCCCATCTACGAAGGTT
	SeActRTR	AGCGGTGGTGGTGAAAGAG
Construction of prokaryotic expression vector	SeRISPOF	CCG *GAGCTC* ATGACTTCGGTCACAAGGGCT
	SeRISPOR	CGG *CTCGAG* TTAACCTACAACTAGCAGGCC
	SlRISPOF	CCG *GAGCTC* ATGACTTCGGTCACAAGGGCT
	SlRISPOD	CGG *CTCGAG* TTAACCTACGACTAACAGGCT
	PxRISPOF	CCG *GAGCTC* ATGACTTCTGTTGTTGTGAGG
	PxRISPOR	CGG *CTCGAG* TTAACCTACAACTAGGGTTCC
	BmRISPOF	CCG *GAGCTC* ATGAATTCTGTGGTAAGAGCT
	BmRISPOR	CGG *CTCGAG* TTAACCGACAACTAACAAGCC
dsRNA synthesis	SeRISPT7F	TAATACGACTCACTATAGGTCGCGGACCAAGTCATGTTCG
	SeRISPR	TTAACCTACAACTAGCAGGCCGTCT
	SeRISPF	TCGCGGACCAAGTCATGTTCGTTTC
	SeRISPT7R	TAATACGACTCACTATAGGTTAACCTACAACTAGCAGGCC
	EGFPT7F	TAATACGACTCACTATAGGCGACGTAAACGGCCACAAGTT
	EGFPR	TTATCTAGATCCGGTGGATCC
	EGFPF	CGACGTAAACGGCCACAAGTT
	EGFPT7R	TAATACGACTCACTATAGGTTATCTAGATCCGGTGGATCC

Specific primers SeRISP3EF1, SeRISP3EF2, SeRISP5ER1, SeRISP5ER2, and SeRISP5ER3 (Table 1) were designed according to the partial sequence cloned above and then used for the rapid amplification of cDNA ends (RACE) for *SeRISP*. In order to amplify the 3′ end and in accordance with the manufacturer's instructions of the 3′-Full RACE Core Set Ver. 2.0 (Takara, Dalian, China), the first round PCR was carried out using SeRISP3EF1 and an Outer Primer using the following protocol: 94°C for 3 min, 30 cycles of 94°C for 30 s, 55°C for 30 s, 72°C for 2 min and a final polymerization step at 72°C for 10 min. The second round PCR was carried out using SeRISP3EF2 and an Inner Primer using the first round PCR product as a template. The thermal cycling protocol was set as follows: 94°C for 3 min, 30 cycles of 94°C for 30 s, 60°C for 30 s, 72°C for 2 min and a final polymerization step at 72°C for 10 min. In order to amplify the 5′end, the 5′ RACE cDNA of *S. exigua* was synthetized using the SeRISP5ER1 primer in accordance with the manufacturer's instructions for the 5′ RACE System for Rapid Amplification of cDNA Ends, Version 2.0 (Invitrogen). First round PCR was carried out using SeRISP5ER2 and an Abridged Anchor Primer with the following protocol: 94°C for 2 min, 30 cycles of 94°C for 30 s, 55°C for 30 s, 72°C for 1 min and a final polymerization step at 72°C for 10 min. The second round PCR was carried out using SeRISP5ER3 and a UAUP Primer, using the first round PCR product as a template. The thermal cycling protocol was set as follows: 94°C for 2 min, 30 cycles of 94°C for 30 s, 60°C for 30 s, 72°C for 1 min; additional polymerization step was set at 72°C for 7 min. Products arising from 3′ RACE and 5′ RACE were finally purified from 2.0% agarose gels, ligated into the T-vector (TaKaRa, Dalian, China) and then sequenced.

### Sequence and data analysis

Sequence similarity and the conserved domains of RISPs were analyzed using BLAST programs on the National Center for Biotechnology Information (NCBI). cDNA sequences and deduced amino acid sequences were analyzed using DNASTAR 7.0. Signal peptide and transmembrane domains were predicted using MEMSAT3 & MEMSAT-SVM (http://bioinf.cs.ucl.ac.uk/psipred/). Functional regions were predicted by Pfam 26.0 (http://pfam.sanger.ac.uk/). Multiple sequence alignments were performed using Multalin version 5.4.1 (http://multalin.toulouse.inra.fr/multalin/multalin.html) and the rare codons were analyzed by graphical codon usage analyzer (http://www.gcua.schoedl.de/) (Thangadurai et al., 2008).

### Quantitative real-time PCR

Total RNAs was extracted from *S. exigua* as described above. cDNAs were then synthesized for quantitative real-time PCR (qRT-PCR) using the PrimeScript RT reagent Kit with gDNA Eraser (TaKaRa, Dalian, China) in accordance with the manufacturer's instructions. cDNAs were diluted 10 times to perform PCR for expression level analysis, or qRT-PCR for expression pattern analysis. The 25 μL reaction volume consisted of 1 μL of cDNA, 12.5 μL of SYBR Green (TaKaRa, Dalian, China), 10.5 μL of ddH_2_O, 0.5 μL of forward primer (10 μM) and 0.5 μL of reverse primer (10 μM). All reactions were performed on a BIO-RAD CFX96 Real-Time PCR Detection System (Bio-Rad, Hercules, CA) in accordance with the manufacturer's recommendations. The optimized real-time PCR program was 94°C for 30 s, followed by 40 cycles of 95°C for 10 s, 65°C for 30 s and 72°C for 15 s. After the cycling protocol, melting curves were obtained by increasing the temperature from 70° to 95°C (0.4°C/s) to denature the double-stranded DNA. Primers for *SeRISP* (SeRISPRTF and SeRISPRTR) and for *S. exigua* β-actin protein (SeActRTF and SeActRTR) (Table 1) were designed using Primer 5.0. Relative quantification (RQ) of *SeRISP* expression was calculated by the 2^−ΔΔ*Ct*^ method (Livak and Schmittgen, 2001). To determine the experimental efficiency of our PCR, we amplified diluted cDNAs (10^−4^, 10^−5^, 10^−6^, 10^−7^, 10^−8^) by real-time PCR. We then created plots of the log of the template concentration vs. the CT. PCR efficiency was then calculated from the slope of the line using the equation, *E* = 10^−1/slope^. All results are presented as mean ± standard error of the mean.

### Prokaryotic expression of recombinant protein and western-blotting

To verify the immunocompetence between homologous RISP proteins and PxRISP antibody to, *in vivo* and prokaryotic protein of RISPs from *S. exigua, S. litura, P. xylostella*, and *B. mori* were exposed to PxRISP via Western-blotting.

Total RNA from the third instar larva of *S. exigua, S. litura, P. xylostella*, and *B. mori* were isolated and the cDNAs synthesized as described above. For each insect, the open reading flame (ORF) of RISPs was cloned from the appropriate cDNA by using a specific set of primers: SeRISPOF, SeRISPOR, SlRISPOF, SlRISPOR, PxRISPOF, PxRISPOR, BmRISPOF, and BmRISPOR (Table 1). Each of these primers featured a *Sac I* (*GAGCTC*) restriction site on one end and a *Xho I* (*CTCGAG*) restriction site on the other end. PCR products from RISPs PCRs, and the pET32a expression vectors, were separately digested using *Sac I* and *Xho I* (New England BioLabs). Then, 50 μL digestion reactions were set up consisting of 2 μg of DNA, 5 μL of 10 × NEB buffer 4, 1 μL of 100 × BSA and 1 μL of 20 U/μL enzyme; these were incubated at 37°C for 4 h. Digested RISPs and pET32a expression vectors were then purified using a Universal DNA Purification Kit (TIANGEN) in accordance with the manufacturer's recommendations and then ligated together using T4 DNA Ligase (New England BioLabs). The 10 μL ligation reaction consisted of 5 μL of purified RISPs, 3 μL of purified vector, 1 μL of 10 × T4 DNA ligase buffer, and 1 μL of T4 DNA ligase. Then, the reaction tubes were incubated at 16°C for 12 h. Recombinant plasmids (pET32a-RISPs) were then transformed into Escherichia coli Transetta (DE3), using a non-carrier pET32a vector as a negative control. Positive recombinant *E. coli* were sequenced and recombinant plasmids extracted using a TIANprep Mini Plasmid Kit (TIANGEN) in order to verify sequence identity and successful ligation.

Twelve hours after activation, the recombinant Transetta (DE3) cells were inoculated into 50 mL of lysogeny broth containing 100 μg/mL of Ampicillin. Cells were then cultured until the OD_600_ lay between 0.6 and 0.8. Then, 1 mmol/L of IPTG was added and the culture allowed continuing growing at 28°C for 6 h to induce the expression of prokaryotic protein. Three mL of bacteria from each sample was then collected and centrifuged at 6,000 × g. The bacterial pellets were then resuspended in 500 μL of 1 × TE Buffer (10 mM of Tris-HCl and 1 mM of EDTA) and lysed by ultrasonic waves. The samples were then centrifuged at 12,000 × g for 10 min at 4°C. The supernatant was transferred into a new tube, while the pellet was resuspended in 500 μL of 1 × TE Buffer. Next, 200 μL of 5 × SDS-PAGE loading buffer was added to tubes containing the pellet or the supernatant and boiled for 5 min in a water bath in order to denature proteins. SDS-PAGE (12%) was used to separate the extracted proteins and determine the molecular mass and expression level of the target recombinant fusion protein (RISPs). Proteins separated by SDS-PAGE were visualized using Coomassie Brilliant Blue.

Next, we aimed to determine the immunoreactivity of RISP fusion protein across different species of insect (*S. exigua, S. litura, P. xylostella*, and *B. mori*). In brief, a PVDF membrane (BioRad) was cut to the size of the gel and pre-soaked in 100% methanol (MeOH). The gel, sponge pads, four pieces of Whatman filter paper and MeOH-soaked membrane were then soaked in transfer buffer (1 × SDS-PAGE Running Buffer, 20% MeOH) for 10 min prior to transfer at 80 mA for 90 min. After transfer, membranes were first exposed to a rabbit antibody against RISP from *P. xylostella* (Gong et al., 2011) at a dilution of 1:2,000 and then exposed to a HRP-labeling sheep secondary antibody against rabbit IgG at a dilution of 1:10,000 (SIGMA). A rabbit antibody against His-Tag was used as a positive control. Positive binding was visualized using a HRP-DAB coloration kit (TIANGEN).

In order to investigate the immunoreactivity of RISPs *in vivo* protein, we extracted total proteins from *S. exigua, S. litura, P. xylostella*, and *B. mori* using the ProteoExtract Complete Protein Extraction Kit (CALBIOCHEM) in accordance with the manufacturer's instructions. Extracted *in vivo* RISPs were separated by SDS-PAGE and immunoblotted using an antibody raised against PxARISP, as described above. A rabbit antibody against β-actin was used as a positive control.

### RNA interference

#### dsRNA synthesis

SeRISPT7F, SeRISPR, SeRISPF, and SeRISPT7R primers (Table 1) were specifically designed to the function region of *SeRISP*; these were designed to synthesize DNA templates with upstream with downstream T7 promoter sequences (under-lined). For control experiments, with non-specific *S. exigua* dsRNA, two DNA templates were cloned from the enhanced green fluorescent protein (EGFP) sequence contained within the eukaryotic expression vector pEGFP-C1 (GenBank Accession Number: U55763.1) and elongated with T7 promoter sites (Meyering-Vos and Muller, 2007) using EGFPT7F, EGFPR, EGFPF and EGFPT7R primers (Table 1). PCRs were conducted to yield dsRNA, followed by the generation of dsRNA with the T7 RiboMax Express RNAi System (Promega), which involved both DNase digestion and dsRNA purification. dsRNA was quantified using a nucleic acid and protein determinator (Eppendorf). Finally, dsRNAs were stored at −20°C for longer storage.

#### dsRNA feeding

We delivered dsRNA to second instar *S. exigua* larvae by simple oral feeding. This was a very efficient and convenient method because it is not invasive (no mortality), and because larvae can be immediately used for subsequent toxicity analysis, which needs to be done before pupation. Larvae were reared in cell culture plates. The artificial diet was prepared by adding dsRNA targeting *SeRISP* (*SeRISP*-dsRNA) at concentration of 4 or 2 μg/μL, while the diets with dsRNA targeting *EGFP* (*EGFP*-dsRNA) was at a concentration of 4 μg/μL; DEPC water used as a negative control. Equal amount of diet (5 g) was added on every individual pellet to ensure equal amount of dsRNA, and the diet was renewed every 12 h. Each treatment consisted of six larvae and was replicated three times. During the process of 96 h dsRNA feeding, the RNAi efficiency was examined every 24 h by qRT-PCR, and after 96 h of feeding, the larvae were changed to an untreated artificial diet. Thus, larvae were allowed to feed for 96 h at 26 ± 1°C and 70 ± 10% relative humidity under a 16:8 h (light:dark) photoperiod.

#### Validation of RNA interference

qRT-PCR was used to validate the effect of gene silencing. Insects were randomly selected from each treatment at each time point (24, 48, 72, and 96 h) after been fed dsRNA. For every treatment, one larva was selected and considered as one sample (0.005 g), and replicated for three times for each treatment. Total RNAs were then isolated and cDNAs synthesized as a template for the detection of *SeRISP* transcripts. The process of qRT-PCR was performed as described in section Quantitative Real-Time PCR. Western-blotting was performed to detect the diversity of SeRISP content in larvae from each treatment and control sample. Total protein was extracted and Western blotting carried out with PxRISP antibody, as described in section Prokaryotic Expression of Recombinant Protein and Western-Blotting.

#### Functional analysis of SeRISP after gene silencing

ATP content was measured after silencing the expression levels of *SeRISP*. ATP content was measured by a luciferin-luciferase method, in accordance with the protocol described in an ATP detection kit (Beyotime, China). In brief, samples (one larva ~2 mg was used as one sample) from different treatment groups were collected on ice and immediately ground with 200 μL of lysis buffer. Three replicates were performed. Samples were then centrifuged at 12,000 × g for 10 min at 4°C and the supernatant was transferred into a new 1.5 mL microcentrifuge tube for ATP measurement. All samples were diluted at a ratio of 1:100 with ATP detection buffer. Luminescence from a 100 μL diluted sample was then assayed in a luminometer (Wallac1420, PerkinElmer, Finland). A standard curve of ATP concentrations was prepared from series of known concentrations (0.01–5 μmol/L) to facilitate calculations and all samples were carried out in triplicate. Values reported herein represent the means ± SD of three replicates (Yang et al., 2002).

The weight and body length of each treated and control larvae were measured 24, 48, 72, and 96 h after dsRNA feeding to determine whether *SeRISP* silencing suppressed larval growth. The pupation rate of 36 randomly-selected larvae from each treatment was recorded 96 h after dsRNA feeding, and the mean pupal weight of 12 randomly-selected pupae from each treatment was recorded at 24 h after pupation to investigate how the silencing of *SeRISP* suppressed pupation. Controlled mating experiments were also performed with adult insects developed from each dose of dsRNA-treated larvae and negative controls. Ten male and ten female larvae were released into each mating jar and fed with 20% honey solution without dsRNA. The total numbers of eggs and larvae that hatched from these eggs were then calculated to determine how the silencing of *SeRISP* could suppress fecundity.

## Results

### Analysis of the full-length *SeRISP* cDNA and deduced amino acid sequence

A 702 bp fragment was amplified from the cDNA of *S. exigua* using the degenerate primers SeRISPDF and SeRISPDR. Based on the sequence of this cDNA fragment, we then successfully amplified the 5′ end (391 bp) and the 3′ end (320 bp) by 5′ RACE and 3′ RACE techniques, respectively. These sequences were then analyzed using the EditSeq function of DNASTART 7.1, and BLAST on NCBI, to ultimately derive the complete sequence of *SeRISP*. Our results demonstrated that the full sequence of *SeRISP* shared 96.0% identity with the RISP gene from *S. litura* at the amino acid level (GenBank Accession Number: HQ599193.1). We called this sequence *SeRISP* and submitted to GenBank (Accession Number: JN992290). The full length *SeRISP* is 1036 bp in length and consists of a 102 bp 5′ end untranslated region, a 118 bp 3′end untranslated region and an 816 bp open reading frame (ORF) (Figure S1). The ORF of *SeRISP* encodes a protein of 271 amino acids with a computed molecular mass of 29.12 kDa and a predicted isoelectric point of 9.02.

Analysis of the amino acid sequence of SeRISP showed that it contains a putative N-terminal signal peptide, a transmembrane domain, and two highly conserved dicysteine-loops (Cys-loop). Multiple alignments of SeRISP amino acid sequences indicated that these were highly conserved across different insect species (Figures S2, S3, Table S1). Among Lepidoptera, the amino acid sequence of *SeRISP* showed the highest conversation with *S. litura* (96.0%), *B. mori* (83.1%), and *P. xylostella* (80.1%). In contrast, *SeRISP* showed relatively low identity among other insects: 63.8, 61.3, 61.0, 63.8, 60.0, 65.1, and 63.6% for *Locusta migratoria, T. castaneum, G. atropunctata, A. aegypti, A. gambiae, C. quinquefasciatus*, and *D. melanogaster*, respectively. Comparing homological RISP amino acid sequences across different insects, we can conclude that the highly conserved region was mainly located at the C-terminal and represented the functional domain of RISP. In particular, the putative ligands for the 2Fe-2S cluster were very highly conserved across different insects. The region encoding the RISP transmembrane domain also was highly conserved across different insects, while the N-terminal signal peptide of SeRISP was only conserved within Lepidoptera. These results demonstrate that RISP is highly conserved in insects from the Lepidoptera and have a similar protein structure; in turn, this implies that these also have similar antigenic determinants.

### The expression pattern of *SeRISP* in different developmental stages

**Figure 1:**
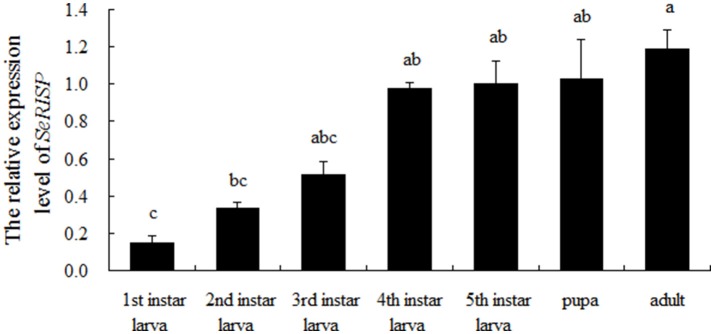
qRT-PCR analysis of *SeRISP* in different developmental stages of *S. exigua*. The expression level was normalized to the expression level of internal reference gene (actin). Data are presented as means ± SE for three experimental replicates. Letters above each bar indicate significant difference in the different stages by Duncan's Multiple Ranges Test (*P* < 0.05).

The expression pattern of *SeRISP* in different developmental stages was analyzed by qRT-PCR. Our data demonstrated that *SeRISP* was expressed in all life stages of *S. exigua*, but there were some differences in the expression patterns across different developmental stages. The relative expression level of *SeRISP* in the first instar was the lowest compared with other developmental stages; *SeRISP* was 2.21-, 3.41-, 6.50-, 6.64-, 6.84-, and 7.87-fold higher in the second instar, third instar, fourth instar, fifth instar, pupa, and adult than in the first instar, respectively (Figure 1). This indicated that the expression level of *SeRISP* increased with larva growth, remained stable during the developmental stages from fourth instar to pupa and finally reached a peak in the adult stage.

### Verification of RNAi at mRNA and protein level

**Figure 2:**
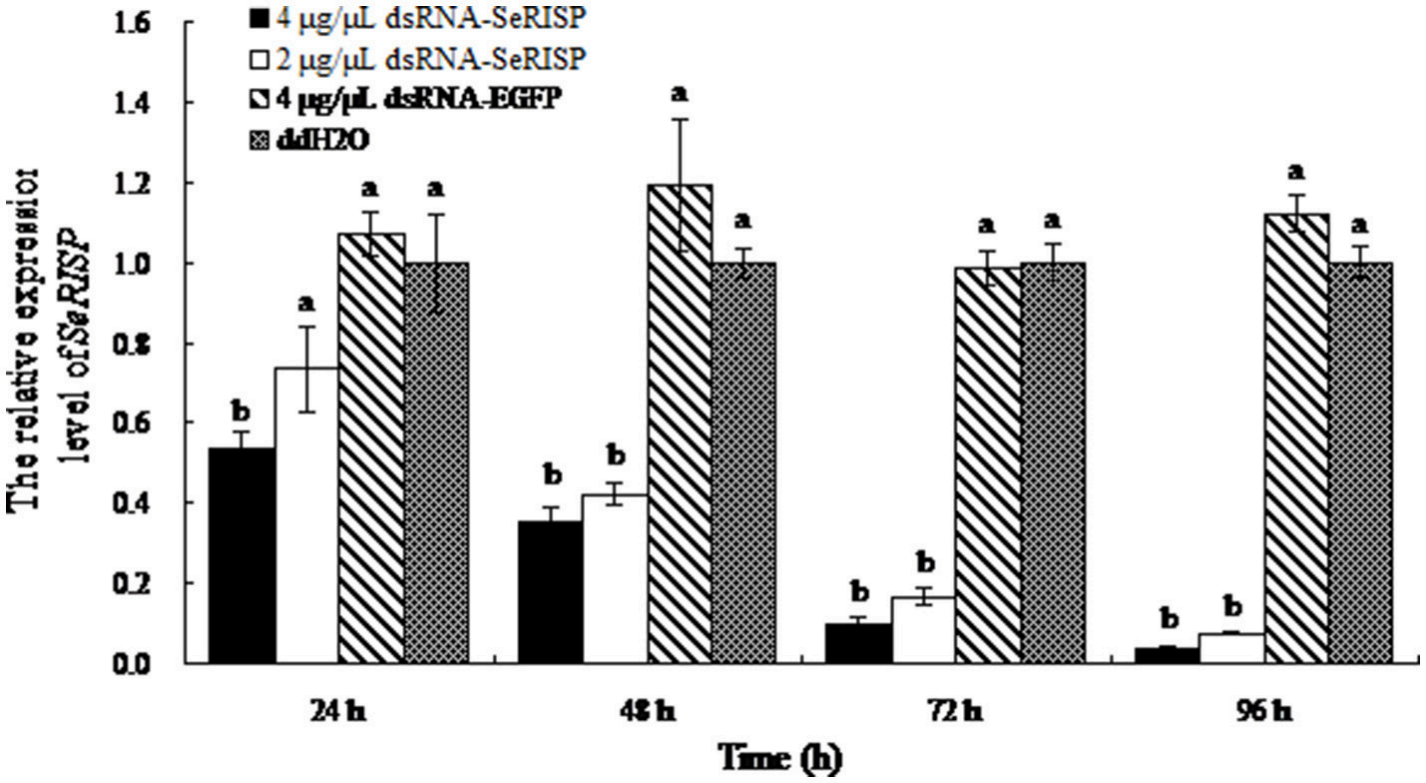
qRT-PCR analysis of transcript levels of *SeRISP* in larvae fed with dsRNA. The expression levels of *SeRISP* in the ddH_2_O treatments were considered as 1 in each group, and expression level of *SeRISP* in other dsRNA treatments were calculated accordingly. Data are presented as means ± SE for three experimental replicates. Letters above each bar indicate significant difference in the different treatments by Duncan's Multiple Ranges Test (*P* < 0.05).

As non-invasive method was applied, the survival rate was 100% in three groups (dsRNA targeting RISP, dsRNA targeting EGFP and DEPC water). Following the dsRNA feeding assay, we monitored mRNA levels of *SeRISP* in larvae using RT-PCR. When larvae were fed with dsRNA-*SeRISP* at a dose of 4 and 2 μg/μL, the silencing rates of *SeRISP* expression were 46 and 27% at 24 h, 65 and 58% at 48 h, 90.3 and 83.5% at 72 h and 96.3 and 92.4% at 96 h compared with each ddH_2_O treatment, respectively. In the control larvae, which were fed with dsRNA-*EGFP* at a dose of 4 μg/μL, the suppression rates were −7, −19, −1.6, and −12.2% at 24, 48, 72, and 96 h, compared with each treatment, respectively (Figure 2). These results indicated that the expression levels of *SeRISP* were ultimately suppressed by dsRNA-*SeRISP*.

**Figure 3:**
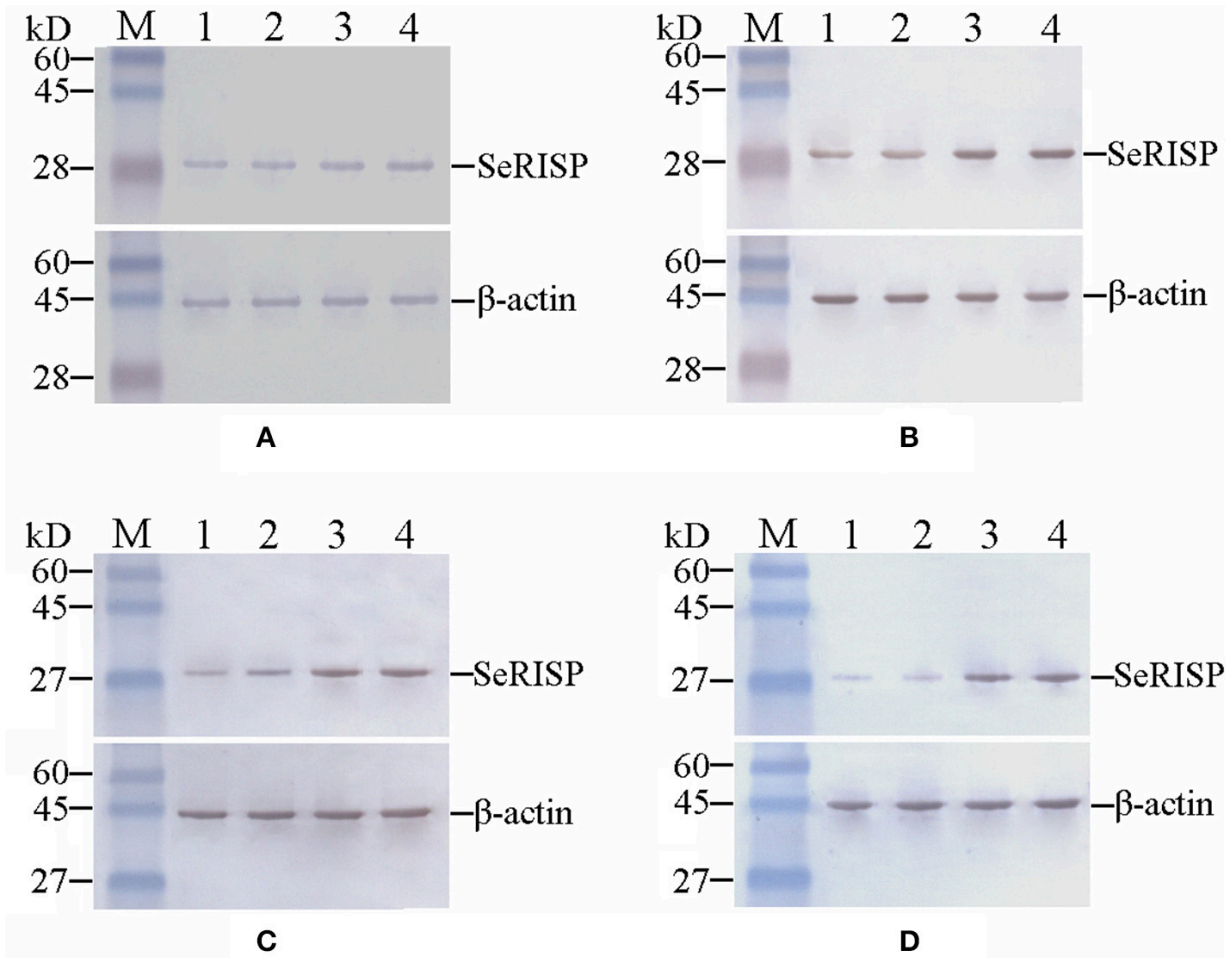
Western-blot analysis of SeRISP protein in larvae fed with dsRNA. **(A–D)**: 24, 48, 72, and 96 h after dsRNA feeding, respectively. Lane M, Protein molecular weight marker; Lane 1, 4 μg/μL dsRNA-*SeRISP*; Lane 2, 2 μg/μL dsRNA-*SeRISP*; Lane 3, 4 μg/μL dsRNA-*EGFP*; Lane 4, ddH_2_O.

To confirm the RISP from *S.exigua* could bind to the antibody of RISP from *P.xylostella*, heterologous RISP protein (29 kDa, pET32a*-SeRISP* for *S.exigua*, pET32a*-SlRISP* for *S.litura*, pET32a*-BmRISP* for *B.mori* and pET32a*-PxRISP* for *P.xylostella*) was fused with Trx-Tag, S-Tag and His-Tag (47 kDa when fused) and harvested from the supernatants of lysed bacteria (Figures S3, S4A). And the results indicated that *in vivo* RISP proteins extracted from *S. exigua, S. litura, B. mori*, and *P. xylostella* were all immunoreactive to PxRISP (Figures S4A,B, S5A,B). In order to investigate SeRISP protein levels, we extracted the total protein content from larvae at 24, 48, 72, and 96 h post dsRNA feeding and immunoblotted with PxRISP antibody. Western-blotting revealed that the protein levels of larvae were slightly different at 24 h (decreased by 15.72% for 4 μg/μL and 14.27% for 2 μg/μL) after dsRNA-*SeRISP* feeding compared with the ddH_2_O treatments (Figure 3A, Table S2), and that these differences became gradually more significant at 48 h (decreased by 39.93% for 4 μg/μL and 29.97% for 2 μg/μL) and 72 h (decreased by 39.90% for 4 μg/μL and 28.86% for 2 μg/μL) after dsRNA feeding compared with ddH_2_O treatments (Figures 3B,C, Table S2). There was a significant difference in SeRISP protein level in the larvae which fed with dsRNA-*SeRISP* 96 h (decreased by 62.08% for 4 μg/μL and 54.43% for 2 μg/μL) compared with those fed with ddH_2_O (Figure 3D, Table S2). However, the SeRISP protein levels in insects fed with dsRNA-*EGFP* at a dose of 4 μg/μL showed no obvious differences when compared across different time-points.

### Effects of *SeRISP* silencing

#### ATP content

**Figure 4:**
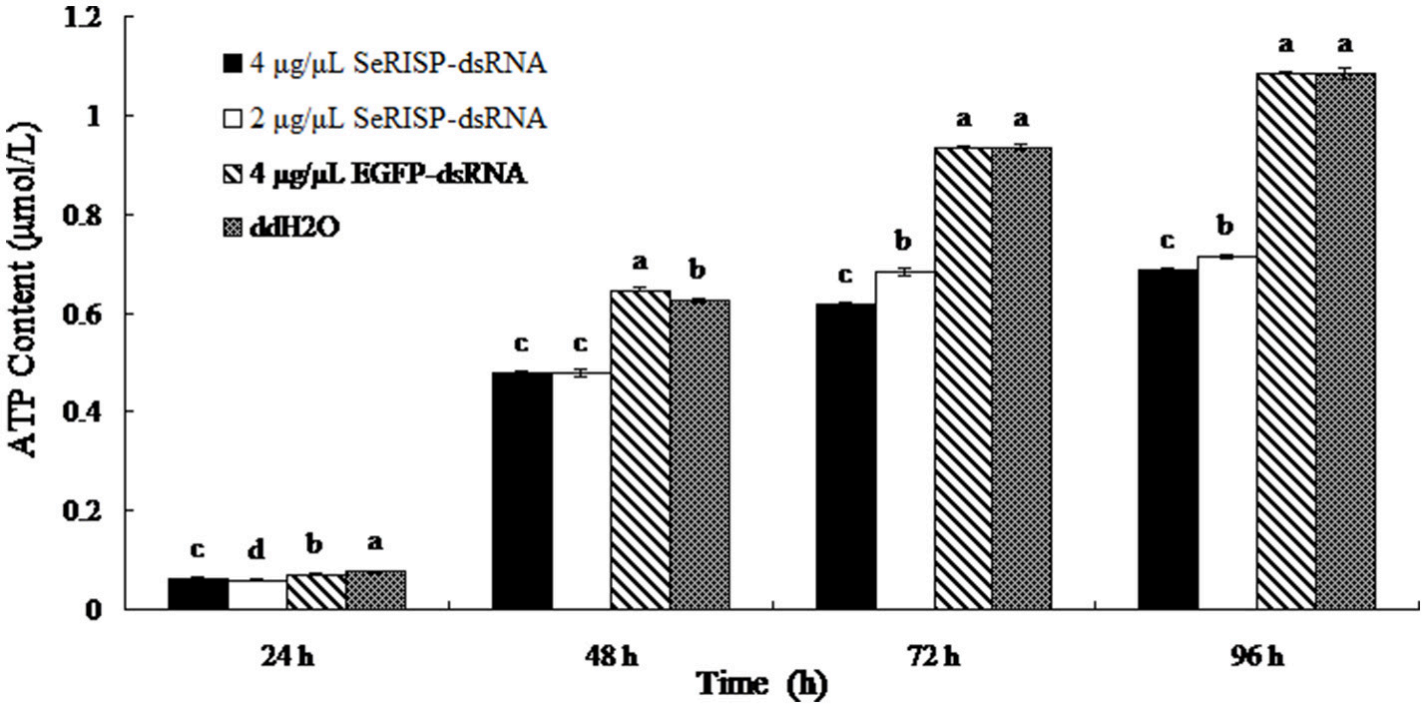
ATP content of larvae fed with dsRNA. Data are presented as means ± SE for three experimental replicates. Letters above each bar indicate significant difference in the different treatments by Duncan's Multiple Ranges Test (*P* < 0.05).

A standard curve of ATP concentration, defined by *y* = 8151.7x + 358.87 (*R*^2^ = 0.9997), was prepared from a series of standard ATP concentrations (0.01, 0.1, 0.5, 1, and 5 μM) as described previously. The ATP contents of larvae were then measured by the luciferin-luciferase method at 24, 48, 76, and 96 h after dsRNA feeding. After feeding larvae with dsRNA-*SeRISP* at a dose of 4 and 2 μg/μL, the suppression rates of larval ATP content were 17.1 and 21.1% at 24 h, 23.7 and 23.7% at 48 h, 33.8 and 26.8% at 72 h and 36.6 and 34.3% at 96 h compared with the ddH_2_O treatment, respectively. While, the larvae fed with dsRNA-*EGFP* at dose of 4 μg/μL, the suppression rates were 6.6, −3.4, 0.2, and −0.2% at 24, 48, 72, and 96 h compared with each treatment, respectively (Figure 4).

#### Larvae weight

**Figure 5:**
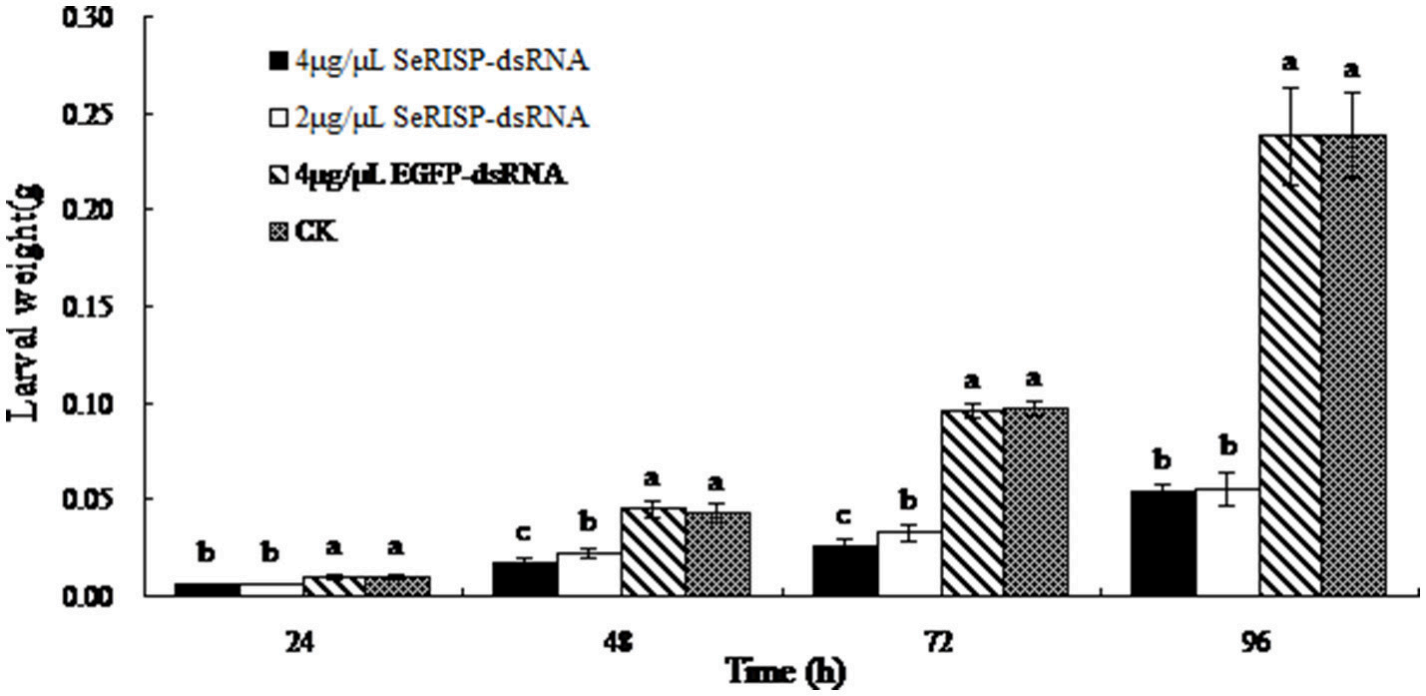
Measurement of larval weight after dsRNA feeding. Data are presented as means ± SE of 12 insects selected randomly. Letters above each bar indicate significant difference in the different treatments by Duncan's Multiple Ranges Test (*P* < 0.05).

**Figure 6:**
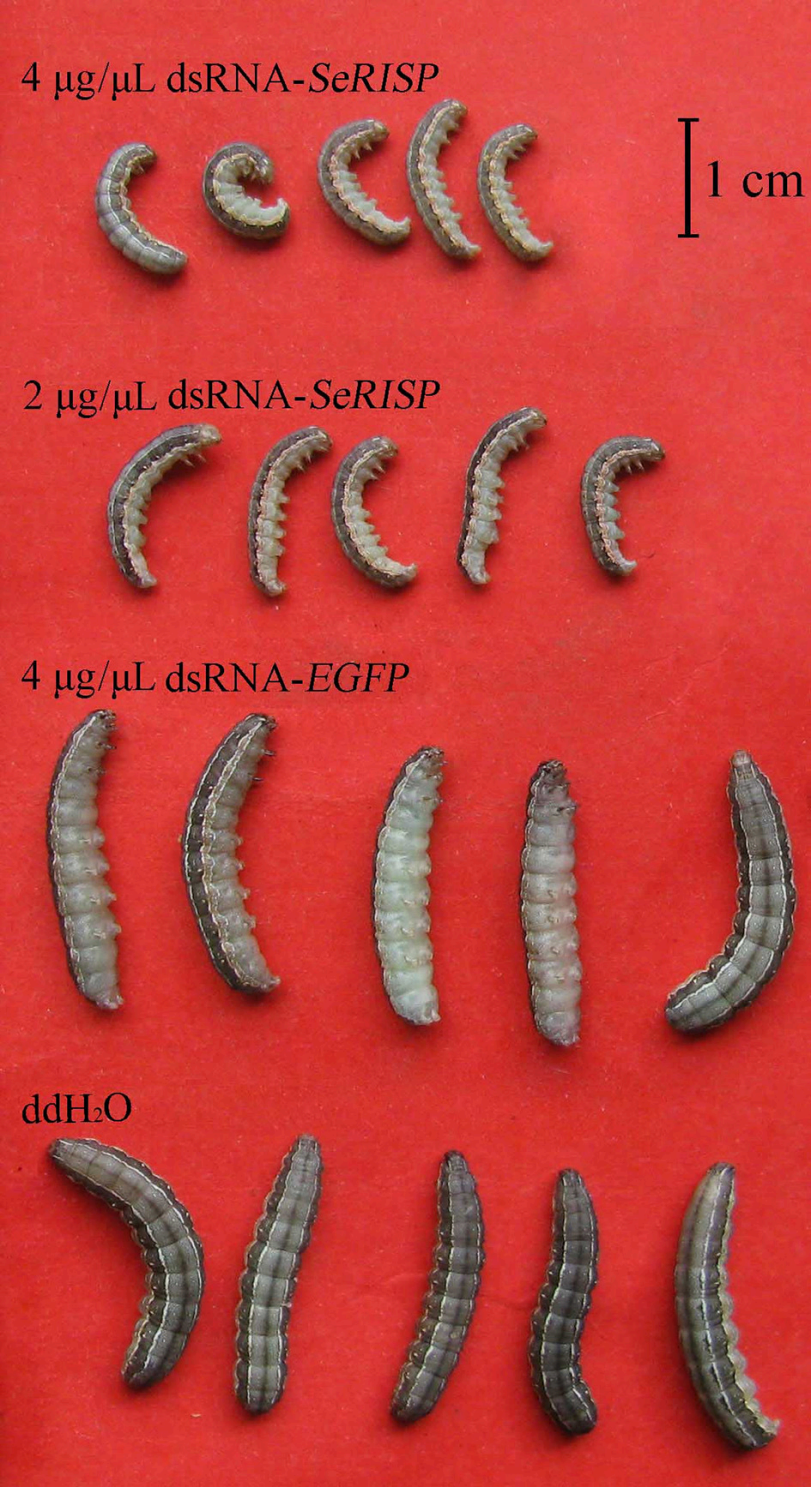
The morphology of 5 insects in each treatment were selected randomly and fainted by CO_2_ after 96 h dsRNA feeding.

The mean weights of larvae were measured at each treatment time following dsRNA treatment process. Results revealed that after the larvae were fed with 4 and 2 μg/μL of dsRNA-*SeRISP*, the mean larval weight was reduced by 43.1 and 40.2% at 24 h, 61.1 and 49.1% at 48 h, 73.1 and 66.3% at 72 h and 77.3 and 76.8% at 96 h compared with the ddH_2_O treatment. Similarly, the mean larval body lengths were also significantly suppressed by dsRNA-*SeRISP* compared with the ddH_2_O treatment at 96 h after dsRNA treatment (Figure 5). However, the larvae fed with dsRNA-*EGFP* at a dose of 4 μg/μL showed suppression rate of 3.9, −3.9, 1.4, and 0.3% at 24, 48, 72, and 96 h compared with ddH_2_O treatment, There were no significant differences in mean body length when compared between the dsRNA-*EGFP* and ddH_2_O treatments (Figure 6).

#### Pupation rate, pupa weight and fecundity of S. exigua

**Figure 7:**
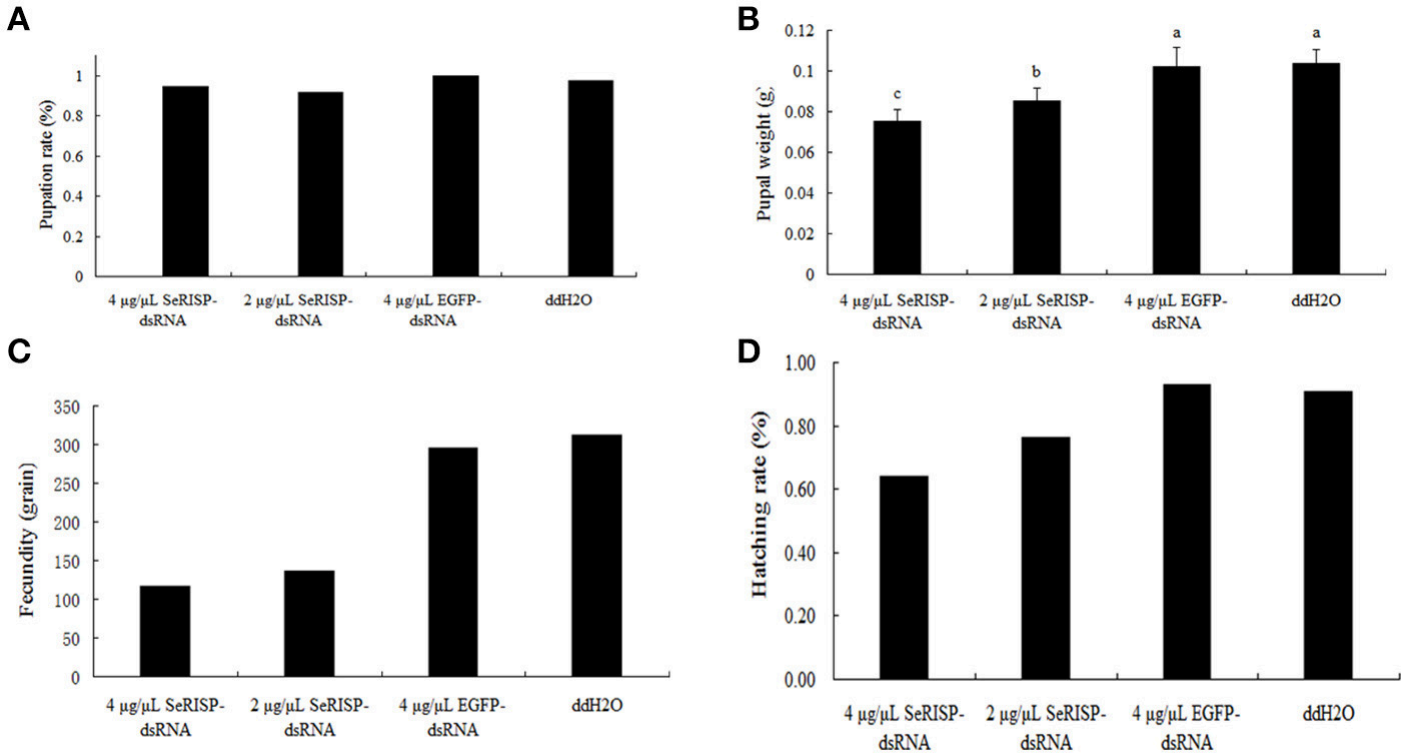
The effects of dsRNA-*SeRISP* on pupal and adult stage after dsRNA feeding. **(A)** The pupation rate of 36 insects selected randomly from the four treatments. **(B)** Mean pupal weight of 12 insects selected randomly from the four treatments. Letters above each bar indicate significant difference in the different treatments by Duncan's Multiple Ranges Test (*P* < 0.05). **(C)** The fecundity laid by 10 male and 10 female adults of *S. exigua* selected randomly from four treatments. **(D)** Hatching rate of the eggs laid by adults from the four treatments as described above.

Ninety six hours after dsRNA treatment process, we provided an artificial diet without dsRNA for the insects. Pupation rates were then measured for the four treatment groups. Pupation rates of 4 μg/μL dsRNA-*SeRISP*, 2 μg/μL dsRNA-*SeRISP*, 4 μg/μL dsRNA-*EGFP* and ddH_2_O treatments were 94.4, 91.7, 100, and 97.22%, respectively (Figure 7A). Pupa weight was measured 24 h after pupation. Results revealed that the mean pupa weight in the groups of 4 μg/μL dsRNA-*SeRISP*, 2 μg/μL dsRNA-*SeRISP*, 4 μg/μL dsRNA-*EGFP* and ddH_2_O treatments were 0.0775, 0.0852, 0.1020, and 0.1036 g, showing that the suppression rates could be reach to 27.1, 17.8, and 1.5% compared with the ddH_2_O treatment, respectively (Figure 7B). We also investigated the fecundity and hatchability of eggs laid by adults fed with dsRNA during the larval stage. Results revealed that the fecundity of larvae fed 4 μg/μL dsRNA-*SeRISP*, 2 μg/μL dsRNA-*SeRISP*, 4 μg/μL dsRNA-*EGFP* and ddH_2_O treatments was 75, 136, 275, and 312 grains, which suggested the suppression rate of fecundity was 62.5, 56.4, and 5.1% compared with ddH_2_O treatment, respectively (Figure 7C). The hatching rate of larva fed 4 μg/μL dsRNA-*SeRISP*, 2 μg/μL dsRNA-*SeRISP*, 4 μg/μL dsRNA-*EGFP* and ddH_2_O treatments was 64.1, 76.5, 92.9, and 90.7%, which suggested the suppression rate of hatchability was 29.3, 15.7, and −2.4% compared with the ddH_2_O treatment (Figure 7D).

## Discussion

Previously analyses of amino acid sequence and protein structure demonstrated that the SeRISP protein can be transported into the mitochondria under the guidance of an the N-terminal signal peptide after modification by the endoplasmic reticulum and is subsequently anchored on the mitochondrial inner membrane with the transmembrane domain (Smith et al., 2017). In the present study, immunoblotting assays demonstrated that the homologous RISP proteins from *S. exigua, S. litura, P. xylostella*, and *B. mori* can be successfully immunoblotted, in both recombinant and *in vivo* forms (Figures S5A,B). The ability to use immunoblotting across homologous proteins can therefore facilitate research of cross-species immunocompetence and promote the development of a commercial antibody for insects.

In this paper, we investigated the expression pattern of *SeRISP* across different developmental stages, which showed an identical expression pattern to the RISP in *P. xylostella* (Gong et al., 2011). Previous RNAi research in insects revealed that the optimal concentration of dsRNA varied across different species (Shukla et al., 2016; Luo et al., 2017). The existence of systemic RNA interference defective gene in *S.exigua* confirmed the presence of the systemic RNAi in this species and enabled the inhibition of gene expression by feeding with dsRNA to non-midgut genes (Tian et al., 2009). In the present paper, two concentrations of dsRNA, 4 μg/μL and 2 μg/μL were fed to the larvae of *S. exigua*. Results demonstrated that the efficiency of this RNAi approach was enhanced as dsRNA concentration increased, and with different treatment times. Similar results were observed in *Helicoverpa armigera* (Xiong et al., 2013; Jin et al., 2015), *Glossina morsitans morsitans* (Attardo et al., 2014), *Acyrthosiphon pisum* (Christiaens et al., 2014; Chen et al., 2016), *Apis mellifera* (Vélez et al., 2016) and *Plutella xylostella* (Han et al., 2014). According to these results, we suggested that the efficiency of RNAi is proportional to the dsRNA concentration within a certain concentration range. Furthermore, in our experiments, the larvae of *S. exigua* were fed with dsRNA at every 12 h and lasted 96 h at maximum; this meant that dsRNA was ingested continuously over this time period and successfully led to gene silencing. Under these circumstances, the silencing efficiencies of *SeRISP* were increased by extending the dsRNA feeding time. The silencing of a target gene can be recovered within an organism after the ingestion of dsRNA digested by RNase III (Terenius et al., 2011), however the continuous ingestion of dsRNA may delay this recovery mechanism and thus enhance the degree of silencing.

RISP is a critical electron transporter of the respiratory chain and pumps protons into the intermembrane space of the mitochondria in order to synthesize ATP (Jafari et al., 2016; Van Vranken et al., 2016). The suppression of RISP at either the mRNA or protein level in insects could therefore have an impact upon ATP synthesis by cutting off the respiratory chain. In this study, the electron transport chain was interrupted successfully *S.exigua* due to the silencing of RISP. This suppressed the process of ATP synthesis and inhibited larvae growth and development due to the insufficient of energy supply. These results are consistent with the silencing effect of RISP in *Trypanosoma brucei* and *Plutella xylostella* by silencing RISP (Smíd et al., 2006; Gong et al., 2011). Furthermore, while larvae growth, pupal weight, fecundity and the hatchability of *S. exigua* were significantly suppressed followed *SeRISP* silencing, there was no significant change in mortality. This may be due to the fact that the vitality of *S. exigua* is greater than that of *Plutella xylostella*, and that the incomplete silencing of *SeRISP* caused by the dsRNA feeding concentration was insufficient to kill the larvae. The effects of RNAi can be maintained in insects for a long period of time. Larvae might not recover immediately after the silence of target gene. In the present study, after 96 h of dsRNA feeding, the larvae could still develop into a pupa, although the pupal weight was smaller than that of the negative controls. Moreover, the fecundity and hatchability of adults were severely suppressed after the silencing of *SeRISP* during the larval period, which may be due to the fact that larvae cannot store a sufficient amount of nutrients for pupation, fecundity and hatchability following the suppression of ATP synthesis. These results further suggest a role for RISP in the process of ATP synthesis. However, further experimentation is now needed to explain the recovery mechanism and enhance the efficiency of silencing. From these results, we can deduce that RISP plays an essential role in the process of ATP synthesis, because it is an important electron transporter in the respiratory chain. The demand for ATP is very low in the initial instars when the insect is small and less active, however, the demand for ATP is increased significantly when the insect begins to eat more and metabolize more rapidly after the third instar stage. The expression level of *SeRISP* was high in the pupal stage, during which the insect is in the process of holometabolous development and requires an abundance of ATP with which to maintain active metabolism. Adults are able to fly, mate and undergo oviposition, so it is this stage of the life cycle where ATP demand is the highest (Reynolds and Hand, 2009); this explains why the expression level of *SeRISP* also peaks in adults.

The results presented here demonstrated that RISP is highly conserved across different species of insects, particularly in Lepidoptera. Based upon our analysis of amino acid sequences, it is evident that the RISP protein has a similar structure in *S. exigua, S. litura* and *B. mori* and can be successfully immunoblotted by an antibody to PxRISP. This might allow us to develop ways of using this cross immunocompetence among homologous proteins. The silencing of RISP by dsRNA feeding proved that it is feasible to suppress the expression of RISP in *S.exigua* by orally feeding dsRNA and revealed the essential role of RISP in the process of ATP synthesis, as well as in the growth and reproduction of *S. exigua*.

## Author contributions

HZ and SS performed the experiments. HZ analyzed the data. XY, LG, and GZ wrote and revised the manuscript.

### Conflict of interest statement

The authors declare that the research was conducted in the absence of any commercial or financial relationships that could be construed as a potential conflict of interest.

## Supplementary material

Figure S1 Nucleotide and deduced amino acid sequence of *SeRISP*. Putative N-terminal signal peptide was underlined, the transmembrane domain was indicated by box, the functional region was marked with shadow and two conserved cysteines to form the dicysteine-loop (Cys-loop) were linked together by black lines.

Figure S2 Multiple alignments of nucleotides of RISP gene in insects. 1, *S. exigua*; 2, *S.litura*; 3, *B*. mori; 4, *P. xylostella*; 5*, L. migratoria*; 6, *T. castaneum*; 7, *G. atropunctata*; 8, *A. aegypti*; 9, *C. quinquefasciatus*; 10, *A. gambiae*; 11, *D. melanogaster*. The dsRNA targeting sequences were highlighted in red box.

Figure S3 Multiple alignments of amino acid sequences deduced from reported RISP gene in insects. The pupative N-terminal signal peptide of SeRISP was indicated by black box, the region of ransmembrane domain was under lined and the putative ligands for the 2Fe-2S cluster were indicated by black spots. GenBank database and there GenBank accession numbers are as follow: *S. litura*, HQ599193.1; *B*. mori, NM001113267.1; *P. xylostella*, EU815629.1; *T. castaneum*, NM001170839.1; *L. migratoria*, GU593056.1; *G. atropunctata*, DQ445508.1; *A. aegypti*, XM001657120.1; *C. quinquefasciatus*, XM001867344.1; *A. gambiae*, XM319708.4; *D. melanogaster*, NM164426.1.

Figure S4 SDS-PAGE analysis of prokaryotic expressed protein of RISPs by using w Transetta (DE3) cells, a type of bacterial strain that replenished six rare codons in *E. coli* (AUA, AGG, AGA, CUA, CCC, and GGA) to express the heterologous protein. **(A)** Sedimentations of crushed bacteria, **(B)** supernatants of crushed bacteria. Lane M, Protein molecular weight marker; lane 1, non-carrier of pET32a; lane 2, pET32a-*SeRISP*; lane 3, pET32a-*SlRISP*; lane 4, pET32a-*BmRISP*; lane 5, pET32a-*PxRISP*.

Figure S5 Immunoblotting assays of RISPs. **(A)** Prokaryotic expressed proteins of RISPs and **(B)** proteins *in vivo* of RISPs. Lane M, Protein molecular weight marker; lane 1, 2, 3, and 4: RISP from *S. exigua, S. litura, B. mori*, and *P. xylostella*.

Table S1 Estimates of evolutionary divergence between sequences.

Table S2 The fluorescence intensity in the western blot to examine the RNAi efficiency.
