# Supplementary figures and images for: Silencing of Rieske Iron-Sulfur Protein Impacts Upon the Development and Reproduction of Spodoptera exigua by Regulating ATP Synthesis
Source: Front Physiol. 2018 May 24;9:575. doi: 10.3389/fphys.2018.00575 (PMC5977497; doi:10.3389/fphys.2018.00575)

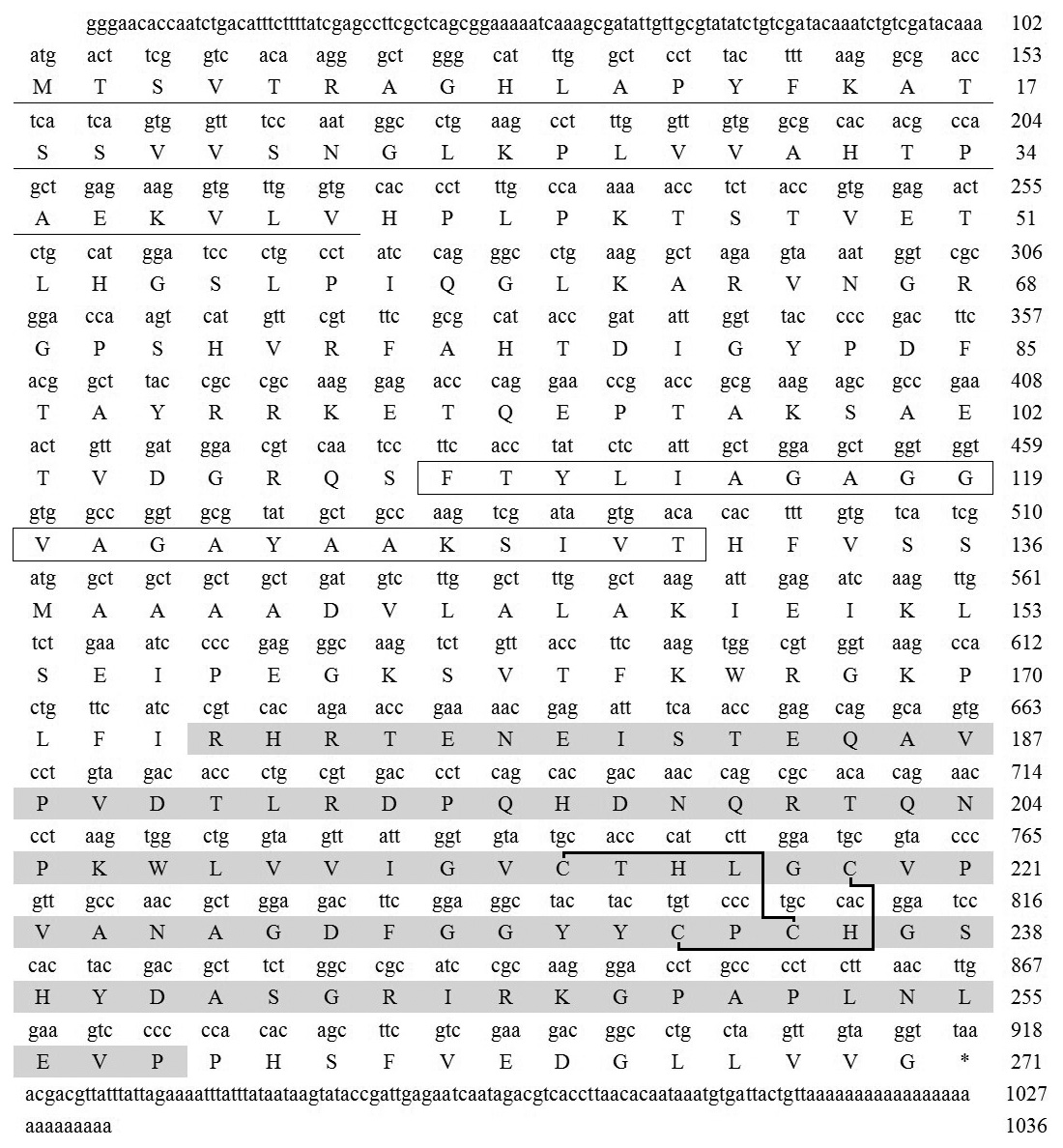

Supplement: Figure S1 — Nucleotide and deduced amino acid sequence of SeRISP. Putative N-terminal signal peptide was underlined, the transmembrane domain was indicated by box, the functional region was marked with shadow and two conserved cysteines to form the dicysteine-loop (Cys-loop) were linked together by black lines. [file Image_1.TIF]

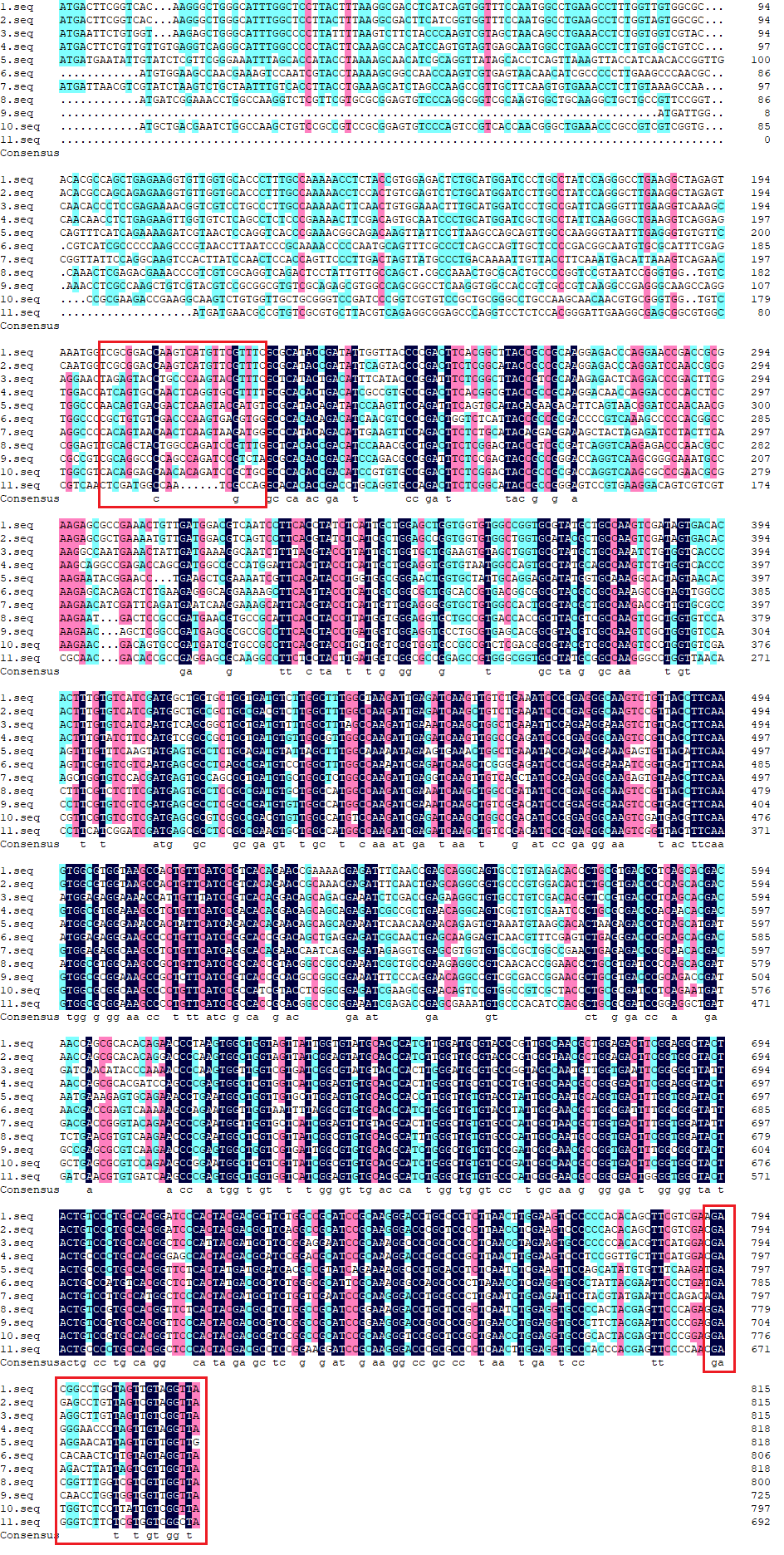

Supplement: Figure S2 — Multiple alignments of nucleotides of RISP gene in insects. 1, S. exigua; 2, S.litura; 3, B. mori; 4, P. xylostella; 5, L. migratoria; 6, T. castaneum; 7, G. atropunctata; 8, A. aegypti; 9, C. quinquefasciatus; 10, A. gambiae; 11, D. melanogaster. The dsRNA targeting sequences were highlighted in red box. [file Image_2.TIF]

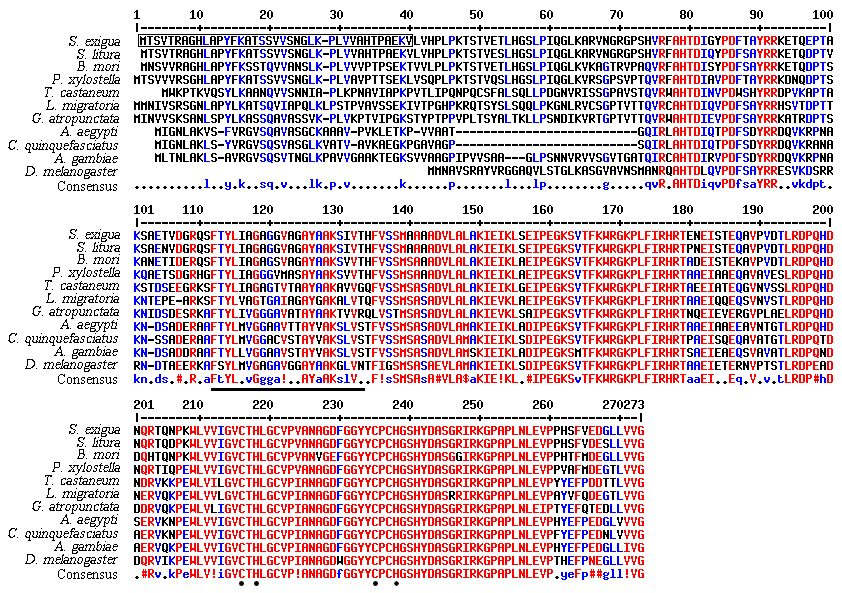

Supplement: Figure S3 — Multiple alignments of amino acid sequences deduced from reported RISP gene in insects. The pupative N-terminal signal peptide of SeRISP was indicated by black box, the region of ransmembrane domain was under lined and the putative ligands for the 2Fe-2S cluster were indicated by black spots. GenBank database and there GenBank accession numbers are as follow: S. litura, HQ599193.1; B. mori, NM001113267.1; P. xylostella, EU815629.1; T. castaneum, NM001170839.1; L. migratoria, GU593056.1; G. atropunctata, DQ445508.1; A. aegypti, XM001657120.1; C. quinquefasciatus, XM001867344.1; A. gambiae, XM319708.4; D. melanogaster, NM164426.1. [file Image_3.TIF]

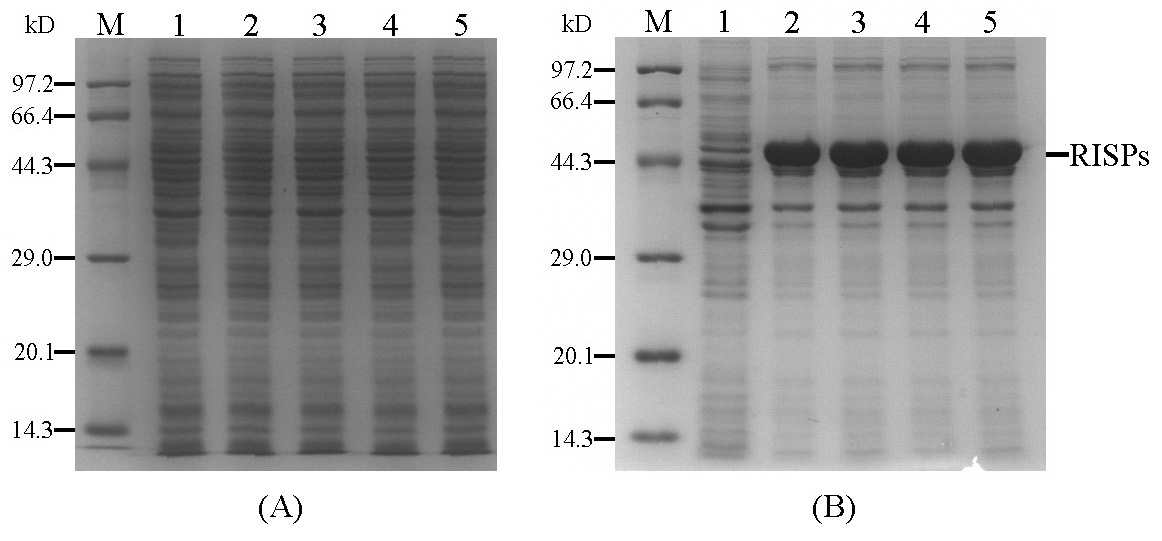

Supplement: Figure S4 — SDS-PAGE analysis of prokaryotic expressed protein of RISPs by using w Transetta (DE3) cells, a type of bacterial strain that replenished six rare codons in E. coli (AUA, AGG, AGA, CUA, CCC, and GGA) to express the heterologous protein. (A) Sedimentations of crushed bacteria, (B) supernatants of crushed bacteria. Lane M, Protein molecular weight marker; lane 1, non-carrier of pET32a; lane 2, pET32a-SeRISP; lane 3, pET32a-SlRISP; lane 4, pET32a-BmRISP; lane 5, pET32a-PxRISP. [file Image_4.TIF]

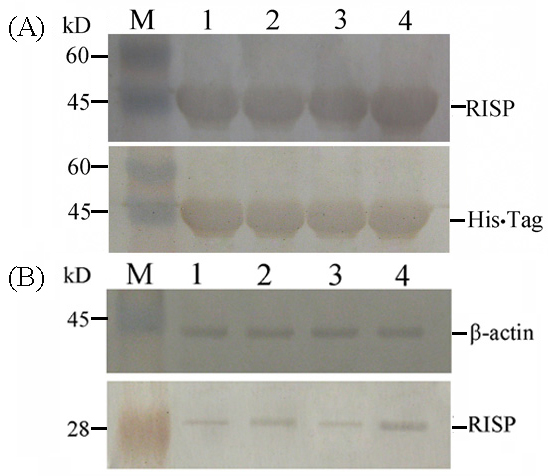

Supplement: Figure S5 — Immunoblotting assays of RISPs. (A) Prokaryotic expressed proteins of RISPs and (B) proteins in vivo of RISPs. Lane M, Protein molecular weight marker; lane 1, 2, 3, and 4: RISP from S. exigua, S. litura, B. mori, and P. xylostella. [file Image_5.TIF]
