# Supplementary material for: Silencing of Rieske Iron-Sulfur Protein Impacts Upon the Development and Reproduction of Spodoptera exigua by Regulating ATP Synthesis
Source: Front Physiol. 2018 May 24;9:575. doi: 10.3389/fphys.2018.00575 (PMC5977497; doi:10.3389/fphys.2018.00575)
Supplement: Table S1 — Estimates of evolutionary divergence between sequences. [file Table_1.docx]

Table S1 Estimates of evolutionary divergence between sequences

The number of amino acid differences per sequence from between sequences are shown. The analysis involved 11 amino acid sequences. All positions containing gaps and missing data were eliminated. There were a total of 206 positions in the final dataset. Evolutionary analyses were conducted in MEGA5.

| Species | A | B | C | D | E | F | G | H | I | J |
| --- | --- | --- | --- | --- | --- | --- | --- | --- | --- | --- |
| *S.exigua* |  |  |  |  |  |  |  |  |  |  |
| *S.litura* | 9.000 |  |  |  |  |  |  |  |  |  |
| *B.mori* | 31.000 | 29.000 |  |  |  |  |  |  |  |  |
| *P.xylostella* | 35.000 | 34.000 | 35.000 |  |  |  |  |  |  |  |
| *L.migratoria* | 59.000 | 54.000 | 55.000 | 47.000 |  |  |  |  |  |  |
| *T.castaneum* | 65.000 | 63.000 | 67.000 | 58.000 | 50.000 |  |  |  |  |  |
| *G.atropunctata* | 68.000 | 65.000 | 61.000 | 60.000 | 48.000 | 57.000 |  |  |  |  |
| *A.aegypti* | 73.000 | 74.000 | 73.000 | 72.000 | 64.000 | 66.000 | 66.000 |  |  |  |
| *C.quinquefasciatus* | 70.000 | 71.000 | 75.000 | 73.000 | 70.000 | 70.000 | 70.000 | 18.000 |  |  |
| *A.gambia* | 72.000 | 74.000 | 76.000 | 70.000 | 64.000 | 68.000 | 67.000 | 30.000 | 30.000 |  |
| *D.melanogaster* | 78.000 | 75.000 | 76.000 | 76.000 | 68.000 | 75.000 | 71.000 | 61.000 | 62.000 | 65.000 |

Table S2 The fluorescence intensity in the western blot to examine the RNAi efficiency

|  | | Intensity (ng) | | | |
| --- | --- | --- | --- | --- | --- |
|  |  | 1 | 2 | 3 | 4 |
| A | SeRISP | 5.3922 | 5.4849 | 6.0616 | 6.3980 |
|  | β-actin | 6.3410 | 6.4308 | 6.4955 | 6.3830 |
| B | SeRISP | 6.8709 | 8.0099 | 11.5500 | 11.4380 |
|  | β-actin | 13.2140 | 12.0000 | 11.2710 | 10.8750 |
| C | SeRISP | 5.1691 | 6.1189 | 7.6341 | 8.6010 |
|  | β-actin | 10.4880 | 10.1810 | 10.4610 | 10.6430 |
| D | SeRISP | 4.1986 | 5.0454 | 10.1640 | 11.0710 |
|  | β-actin | 12.3940 | 14.0290 | 12.8350 | 15.3340 |
